# Antagonism between phytohormone signalling underlies the variation in disease susceptibility of tomato plants under elevated CO_2_

**DOI:** 10.1093/jxb/eru538

**Published:** 2015-02-05

**Authors:** Shuai Zhang, Xin Li, Zenghui Sun, Shujun Shao, Lingfei Hu, Meng Ye, Yanhong Zhou, Xiaojian Xia, Jingquan Yu, Kai Shi

**Affiliations:** ^1^Department of Horticulture, Zijingang Campus, Zhejiang University, 866 Yuhangtang Road, Hangzhou, 310058, P.R. China; ^2^Tea Research Insititute, Chinese Academy of Agricultural Science, Hangzhou, 310008, P.R. China; ^3^Key Laboratory of Horticultural Plants Growth, Development and Quality Improvement, Agricultural Ministry of China, 866 Yuhangtang Road, Hangzhou, 310058, P.R. China; ^4^Institute of Insect Science, College of Agriculture & Biotechnology, Zhejiang University, 866 Yuhangtang Road, Hangzhou, 310058, P.R. China

**Keywords:** *Botrytis cinerea*, nonexpressor of pathogenesis related genes 1 (NPR1), elevated CO_2_, jasmonic acid, plant disease, *Pseudomonas syringae*, salicylic acid, *Solanum lycopersicum* (tomato), tobacco mosaic virus (TMV).

## Abstract

Increasing CO_2_ concentrations ([CO_2_]) have the potential to disrupt plant–pathogen interactions in natural and agricultural ecosystems, but the research in this area has often produced conflicting results. Variations in phytohormone salicylic acid (SA) and jasmonic acid (JA) signalling could be associated with variations in the responses of pathogens to plants grown under elevated [CO_2_]. In this study, interactions between tomato plants and three pathogens with different infection strategies were compared. Elevated [CO_2_] generally favoured SA biosynthesis and signalling but repressed the JA pathway. The exposure of plants to elevated [CO_2_] revealed a lower incidence and severity of disease caused by tobacco mosaic virus (TMV) and by *Pseudomonas syringae*, whereas plant susceptibility to necrotrophic *Botrytis cinerea* increased. The elevated [CO_2_]-induced and basal resistance to TMV and *P. syringae* were completely abolished in plants in which the SA signalling pathway *nonexpressor of pathogenesis-related genes 1* (*NPR1*) had been silenced or in transgenic plants defective in SA biosynthesis. In contrast, under both ambient and elevated [CO_2_], the susceptibility to *B. cinerea* highly increased in plants in which the JA signalling pathway *proteinase inhibitors* (*PI*) gene had been silenced or in a mutant affected in JA biosynthesis. However, plants affected in SA signalling remained less susceptible to this disease. These findings highlight the modulated antagonistic relationship between SA and JA that contributes to the variation in disease susceptibility under elevated [CO_2_]. This information will be critical for investigating how elevated CO_2_ may affect plant defence and the dynamics between plants and pathogens in both agricultural and natural ecosystems.

## Summary

Under elevated CO_2_, interactions between tomato and pathogens with different infection strategies were compared. This work highlights modulated SA/JA cross talk contributes to variation in disease susceptibility under elevated CO_2_.

## Introduction

Global climate change due to increasing anthropogenic emissions is markedly affecting natural ecosystems (Kerr, 2007). Rising CO_2_ levels, among other factors, are thought to be responsible for climate change (IPCC, 2007). Furthermore, concentrations of carbon dioxide ([CO_2_]) have increased markedly since the inception of the industrial revolution, reaching current levels of 380 μmol mol^–1^, and they will continue climbing to 730–1020 μmol mol^–1^ by the end of the twenty-first century (Meehl *et al.*, 2005). Additionally, the rise in CO_2_ is often projected to increase the production and quality of agroecosystems, particularly in C_3_ greenhouse vegetable crops (Uprety, 1998; Dion *et al.*, 2013). Many studies have investigated the likely impacts of rising CO_2_ concentration on crop growth and production (Tubiello *et al.*, 2000; Leakey *et al.*, 2006; Soares *et al.*, 2008; Aranjuelo *et al.*, 2013), and there has been general agreement on the beneficial effects of elevated [CO_2_] on yield, probably due to increased photosynthesis, C:N ratio, and water-use efficiency, from the CO_2_ “fertilization effect” (Drake *et al.*, 1997; Ainsworth and Long, 2005; Slattery *et al.*, 2013). However, yield-limiting factors such as pathogens have been ignored in most of these studies (Pangga *et al.*, 2011; Juroszek and von Tiedemann, 2013). Disease symptoms are influenced by three main components: (i) host, (ii) pathogen, and (iii) environmental conditions (McElrone *et al.*, 2005). Thus, the altered environmental conditions associated with elevated [CO_2_] will potentially modify plant disease susceptibility. However, knowledge of the effects of climate change on diseases and related plant responses is still lacking. Pathogens reduce plant productivity worldwide, and billions of dollars in plant yield are lost to diseases each year. Therefore, more work is needed to elucidate how plant diseases will respond to the interacting factors of elevated [CO_2_] climatic conditions (McElrone *et al.*, 2010; Runion *et al.*, 2010). Understanding such relationships is essential for predicting disease pressure and managing agricultural and natural ecosystems under changing climatic conditions.

Limited research on the influence of elevated [CO_2_] on plant pathogens and diseases shows that the severity and/or incidence of disease may increase, decrease, or remain unaffected (Lake and Wade, 2009; Newton *et al.*, 2011; Pugliese *et al.*, 2011; West *et al.*, 2012). Free-air CO_2_ enrichment (FACE) facilities allow for an assessment of the effects under field conditions. Such studies have found that rice plants grown under elevated [CO_2_] conditions showed an increased susceptibility to both rice blast and sheath blight (Kobayashi *et al.*, 2006), whereas in *Solidago rigida*, the disease incidence of leaf spot was reduced by half under similar FACE conditions (Strengbom and Reich, 2006). Another FACE study investigating crown rot on wheat found that elevated [CO_2_] resulted in increased biomass of the necrotrophic fungal pathogen *Fusarium pseudograminearum* and increased stem browning (Melloy *et al.*, 2010). Climate chamber-based studies also report conflicting results. The anthracnose pathogen *Colletotrichum gloeosporioides* increased in aggressiveness over 25 sequential infection cycles in the host *Stylosanthes scabra* under elevated [CO_2_] (Chakraborty and Datta, 2003). However, investigations into the systemic responses of tomato to tomato yellow leaf curl virus (TYLCV) and of tobacco to potato virus Y found that elevated [CO_2_] decreased disease incidence and severity (Matros *et al.*, 2006; Huang *et al.*, 2012). These FACE and chamber studies support earlier findings that plant disease responses to elevated [CO_2_] vary with the host–pathogen system. In some cases, predictions of higher disease levels have been verified, especially for necrotrophic pathogens (Eastburn *et al.*, 2010; Melloy *et al.*, 2010; Eastburn *et al.*, 2011). In contrast, plant defences against (hemi)biotrophic pathogens, including viruses, were generally more efficient under elevated [CO_2_], although there were exceptions. Plants have evolved complex signalling networks to sense and respond to pathogen attacks, and it is generally accepted that the salicylic acid (SA) signalling pathway is mainly activated in response to biotrophic or hemibiotrophic pathogens, whereas resistance to necrotrophic pathogens requires the activation of the jasmonic acid (JA) signalling pathway, which incorporates ethylene (ET)-dependent responses in some cases (Tsuda and Katagiri, 2010; Xin and He, 2013). It was thus hypothesized that SA/JA cross talk was modulated under elevated [CO_2_], which may create a flexible signalling network that is vital for defence responses to different types of invaders. Indeed, elevated [CO_2_] typically increases the C/N ratio and causes plants to re-allocate resources to synthesize secondary metabolites, leading to a shift in leaf chemistry components (Matros *et al.*, 2006). Previous studies demonstrated that elevated [CO_2_] down-regulated the expression of genes related to the JA pathway in soybeans (Zavala *et al.*, 2008; Zavala *et al.*, 2013). In an investigation into tomato plants, elevated [CO_2_] increased the SA level upon uninfected and TYLCV-infected treatments (Huang *et al.*, 2012). Thus, whether the flexible SA/JA cross talk is associated with the elevated [CO_2_]-induced alteration in plant defence strategies needs to be tested in a biological context using the same system, which might account for the highly specific nature of host–pathogen interactions under elevated [CO_2_].

In this study, the spectrum of plant–pathogen interactions were compared under elevated [CO_2_] using fungal, bacterial, and viral pathogens on tomato plants. These pathogens are common, have a wide host range, cause destructive foliar disease, and are widely distributed throughout the world. They have been widely investigated, and plants primarily defend against them through either SA-dependent basal resistance, as observed with *Pseudomonas syringae* and tobacco mosaic virus (TMV), or JA/ET-dependent basal resistance, as observed with necrotrophic *Botrytis cinerea* (Ton *et al.*, 2002; El Oirdi *et al.*, 2011). Here, it was found that elevated [CO_2_] generally favoured the SA signalling pathway and repressed the JA pathway, which was accompanied by enhanced resistance to *P. syringae* and TMV, and susceptibility to *B. cinerea*. Silencing genes in the SA or JA signalling pathways or using plant lines defective in SA or JA biosynthesis overturned the [CO_2_]-induced resistance or susceptibility. This work highlights SA/JA cross talk in specific host–pathogen interactions under elevated [CO_2_]. This information is important for making proper predictions of disease pressure and for designing strategies to improve plant pathogen resistance under changing agricultural conditions.

## Materials and methods

### Plant material and growth conditions

Tomato (*Solanum lycopersicum* L. cv. Zheza 205) seeds were purchased from the Zhejiang Academy of Agricultural Sciences, China and were sown approximately 0.5cm deep in sterilized soil and germinated at 25 °C. Fifteen days after germination, the seedlings were transplanted into plastic pots (diameter, 10.5cm; depth, 17.5cm; one plant per pot) containing soil and perlite (1:3, v:v) in controlled-environment growth chambers (Conviron, Winnipeg, Canada). The growth conditions were as follows: the photosynthetic photo flux density (PPFD) was 600 μmol m^–2^ s^–1^, the photoperiod was 14/10h (day/night), the day/night air temperature was 26/22 °C, and the relative humidity was 85%. When the seedlings were at the four- to five-leaf stage, they were exposed to atmospheric [CO_2_] at either 380 μmol mol^–1^ or 800 μmol mol^–1^, corresponding to the “ambient [CO_2_]” and “elevated [CO_2_]” treatments, respectively. After 4 d of acclimation, plants exposed to both ambient and elevated [CO_2_] were subjected to inoculation with TMV, *P. syringae*, or *B. cinerea*. Plants were also mock inoculated to control for tissue damage caused by the inoculation procedure. The pot placement within each [CO_2_] condition was randomized every 2 d; all plants were watered and fertilized with Hoagland’s solution every 2–3 d as necessary. The experiments were conducted independently three times.

### Pathogen inoculation

For TMV inoculation, two fully developed leaves were inoculated with TMV (U1 strain) suspensions using cotton tips on adaxial surfaces previously dusted with carborundum powder (Liao *et al.*, 2012).

The bacteria *P. syringae* pv. *tomato* DC3000 were cultured in King’s B medium containing 25mg l^–1^ rifampicin. An overnight culture was diluted 1:50 with fresh King’s B medium before the experiment and grown for another 2h at 28 °C. Bacterial cells were harvested by centrifugation (4 °C, 3000rpm, 10min) and dissolved in 10mM MgCl_2_ to optical density (OD)=0.2 measured at 600nm, which corresponded to approximately 10^8^ colony-forming units (cfu) ml^−1^. Tomato plants were vacuum infiltrated with *P. syringae* suspended in 10mM MgCl_2_ at a final concentration of 10^5^ cfu ml^–1^ after serial dilution according to Katagiri *et al.* (2002). Bacterial leaf populations were measured according to the method described in Wolfe *et al.* (2000). Trypan blue staining was carried out according to Bai *et al.* (2012).

The *B. cinerea* isolate used in this study is BO5-10, and was sub-cultured using the method described in El Oirdi *et al.* (2010). Two different inoculation methods were used in the current study. In the *in planta* inoculation method, all the leaves on the plants were inoculated by spraying them with a *B. cinerea* spore suspension at a density of 2×10^5^ spores per ml. In the *in vitro* inoculation method, detached fully developed leaves were spot inoculated with a *B. cinerea* suspension (2×10^5^ spores per ml) using a 2.5 μl droplet of *B. cinerea* spores on the upper surface of each leaf using a micropipette (El Oirdi *et al.*, 2010). After inoculation, disease symptoms were assessed by trypan blue staining (Bai *et al.* 2012), quantification of *B. cinerea* gene transcription, or by analysis of chlorophyll fluorescence with an Imaging-PAM Chlorophyll Fluorometer (IMAG-MAXI, Heinz Walz, Effeltrich, Germany). For *B. cinerea* actin gene transcription assay, the primers used are shown in Supplementary Table S1. PCR conditions consisted of denaturation at 95 °C for 3min, followed by 40 cycles of denaturation at 95 °C for 10 s, and annealing at 58 °C for 45 s. For the chlorophyll fluorescence assay, in actinic light (300 μmol m^−2^ s^−1^), maximal fluorescence (*F*_m_′), and steady-state fluorescence before the flash (*F*) were measured, whereas saturated light flashes were applied every 20 s, and the quantum efficiency of light-adapted leaves (ΦPSII) was calculated as *F*_m_′–*F*/*F*_m_′ (Genty *et al.*, 1989).

### Virus-induced gene silencing in tomato

Virus-induced gene silencing (VIGS) was performed using the bipartite tobacco rattle virus (TRV) vectors, pTRV1 and pTRV2, as previously described (Liu *et al.*, 2002). Fragments from tomato *nonexpressor of pathogenesis-related genes 1* (*NPR1*), *proteinase inhibitors I* and *II* (*PI I* and *PI II*) cDNAs were PCR-amplified using the primers shown in Supplementary Table S1. Restriction sites were added to the 5’ ends of the forward and reverse primers for cloning into the pTRV2 vector. Amplification using these primers produced a 300-bp fragment. pTRV2 vectors containing the cDNA fragments were also described in El Oirdi *et al.* (2011). The pTRV-RNA2 empty vector (pTRV:0) was used as a control. The resulting plasmids were subsequently introduced into *Agrobacterium tumefaciens* strain GV3101, and a culture of *Agrobacterium tumefaciens* (OD_600_=0.9) containing either the pTRV:0 or the pTRV: target gene and pTRV-RNA1 (OD_600_=0.9) in a 1:1 ratio was infiltrated into fully expanded cotyledons of tomato plants. It should be noted that pTRV:*PI* was an equal mix of pTRV:*PI I* and pTRV:*PI II*. The inoculated plants were grown under a 14-h photoperiod at 22 °C. After 3–4 weeks, the levels of targeted transcripts were analysed by qRT-PCR using the primers in Supplementary Table S1.

### RNA isolation and transcript analysis

Total RNA from tomato leaves was prepared using TRIzol reagent (Invitrogen, Carlsbad, CA, USA) according to the manufacturer’s procedure. Genomic DNA was removed using a purifying column. Reverse transcription was performed using Superscript II (Invitrogen) following the manufacturer’s instructions. The primers are listed in Supplementary Table S1, and most of these primers have been described previously in El Oirdi *et al.* (2011). qRT-PCR analysis was performed using the StepOnePlus Real-Time PCR system (Applied Biosystems, Foster City, CA, USA) with *Power* SYBR Green PCR Master Mix (Applied Biosystems). Gene expression was normalized to actin, and relative gene expression was calculated as described by Livak and Schmittgen (2001). For semi-quantitative RT-PCR analysis of *TMV-coat protein* (*CP*) gene, the PCR reaction was performed using the TaKaRa Ex Taq Hot Start Version (Takara Bio) with denaturing, annealing, and extension at temperatures of 94 °C for 30 s, 55 °C for 30 s, and 72 °C for 1min, respectively. The PCR products were analysed by electrophoresis on a 2% agarose gel, and actin was used as a control.

### SA and JA quantification

Frozen plant material (about 100mg) was homogenized in 2-ml microcentrifuge tubes and then 1ml of ethyl acetate spiked with labelled internal standards (D3-JA and D6-SA) was added to each sample. After centrifugation at 13 000 *g* for 20min at 4 °C, supernatants were transferred to fresh 2ml Eppendorf tubes and then evaporated to dryness on a vacuum concentrator (Eppendorf). The residue was resuspended in 0.5ml of 70% methanol (v/v) and centrifuged for 10min at 4 °C (13 000 *g*). The supernatants were pipetted to glass vials and then analysed by HPLC-MS/MS using the same method as described in Wu *et al.* (2007). Each treatment was biologically replicated five times.

### Statistical analysis

At least four independent replicates were conducted for each determination. The data were subjected to analysis of variance, and the means were compared using Tukey’s test at the 5% level.

## Results

### Effects of elevated [CO_2_] on pathogen incidence and severity

**Fig. 1.**
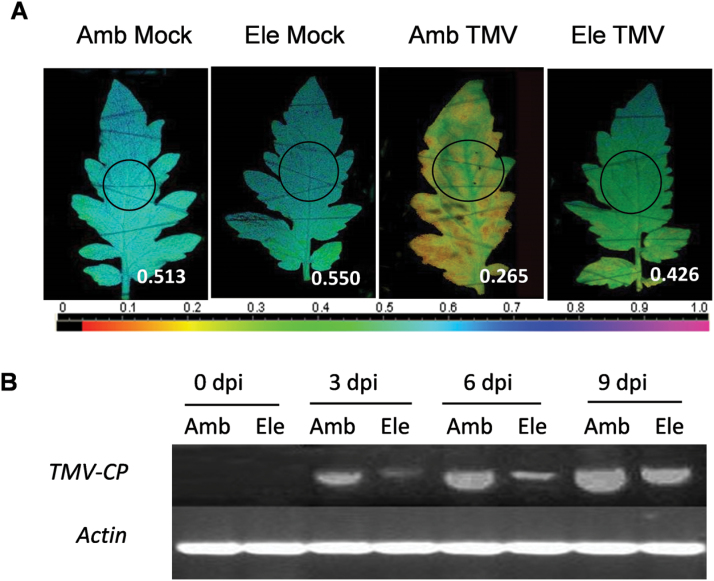
Effects of tobacco mosaic virus infection on tomato plants grown under elevated (Ele, 800 μmol mol^–1^) or ambient [CO_2_] (Amb, 380 μmol mol^–1^). (A) The leaf photochemical quantum yield at photosystem II was measured after 9 d of different treatments. The circles in the images indicate the locations where the fluorescence measurements were performed, and the data are shown in each figure. The colour gradient scale below the figure indicates the magnitude of the fluorescence signal represented by each colour. (B) Time-course changes in the transcription of the gene encoding the TMV-coat protein (CP) in young, fully expanded leaves.

TMV-inoculated plants in ambient and elevated [CO_2_] conditions were compared first. As pathogen infection often results in a reduction in the operating efficiency of PSII, the chlorophyll fluorescence imaging method was used to analyse the response of photochemical quantum yield at photosystem II (ΦPSII) to TMV infection under elevated [CO_2_] (Fig. 1A). TMV infection was significantly reduced under elevated [CO_2_]. At 9 d post-inoculation (dpi), TMV inoculation decreased ΦPSII in the upper uninoculated systemic leaves, whereas ΦPSII remained significantly higher in plants under the elevated [CO_2_] condition compared with those at ambient [CO_2_]. Moreover, the accumulation of *TMV-CP* mRNA detected by semi-quantitative RT-PCR analysis correlated well with the change in ΦPSII (Fig. 1B). All tested uninfected tomato leaves were negative. Among TMV-inoculated plants, transcript levels of the gene encoding the TMV-CP increased steadily from 3–9 dpi in both ambient and elevated [CO_2_]. However, the transcript level was always lower under elevated [CO_2_] than under ambient [CO_2_].

**Fig. 2.**
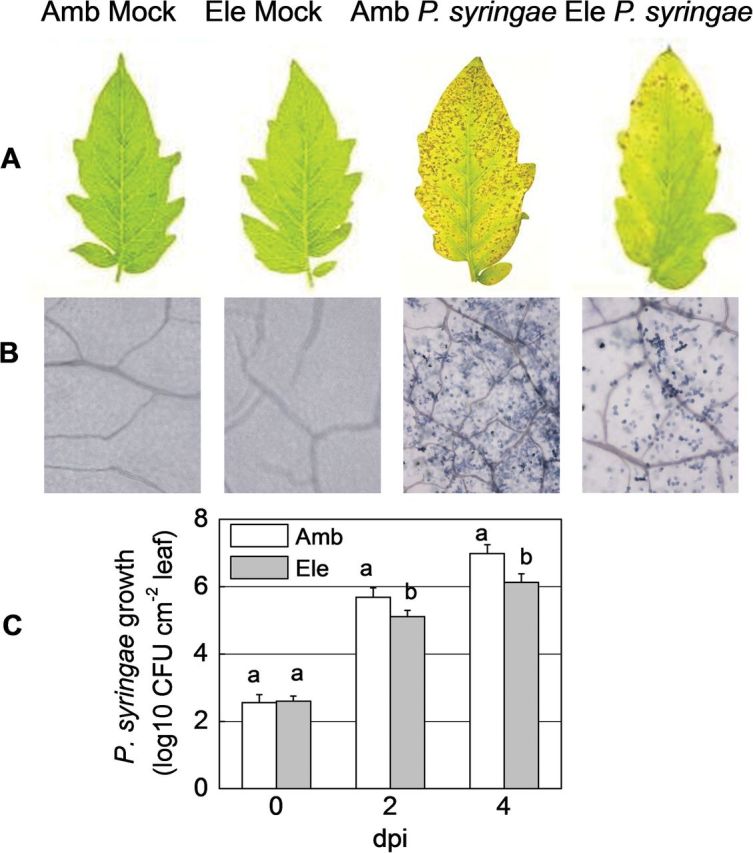
Effects of *Pseudomonas syringae* infection on tomato plants grown under elevated (Ele, 800 μmol mol^–1^) or ambient [CO_2_] (Amb, 380 μmol mol^–1^). (A) Disease symptoms were photographed 6 days post-inoculation. (B) Trypan blue staining for cell death was performed 4 days post-inoculation. (C) *In planta* multiplication of *P. syringae* bacterial populations. The results are expressed as the mean values±SD, *n*=4. Different letters indicate significant differences between the treatments (*P<*0.05).

The effects of CO_2_ enrichment were similar between *P. syringae* and TMV inoculation. Under elevated [CO_2_], treatment of the leaves with bacterial *P. syringae* resulted in a significant reduction of disease symptoms (6 dpi) and cell death (4 dpi) (Fig. 2A, B). Growth analysis of the pathogen also showed that plants grown under elevated [CO_2_] had significantly lower bacterial colony counts at 2 and 4 dpi, compared with plants under ambient [CO_2_] (Fig. 2C).

**Fig. 3.**
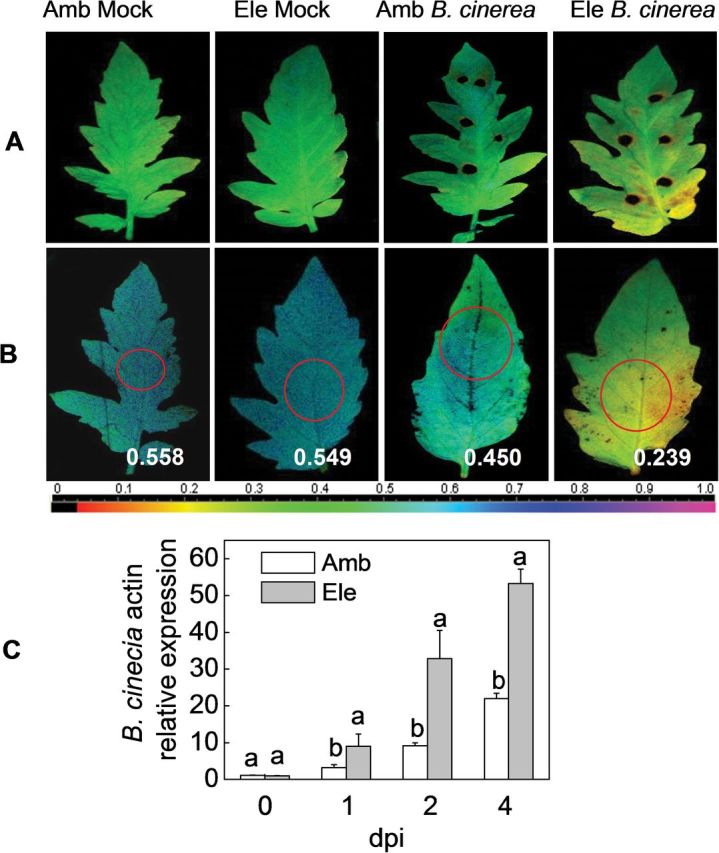
Effects of *Botrytis cinerea* infection on tomato plants grown under elevated (Ele, 800 μmol mol^–1^) or ambient [CO_2_] (Amb, 380 μmol mol^–1^). (A) The leaf photochemical quantum yield at photosystem II (ΦPSII) was measured in the detached leaves after 4 d of *in vitro B. cinerea* inoculation. (B) Leaf ΦPSII was measured after 4 d of *in planta B. cinerea* spray inoculation. The circles in the images indicate the locations where the fluorescence measurements were performed, and the data are shown in each figure. The colour gradient scale below A and B indicates the magnitude of the fluorescence signal represented by each colour. (C) *B. cinerea actin* gene expression after 4 d of *in planta* inoculation in tomato leaves. The results are expressed as the mean values±SD, *n*=4. Different letters indicate significant differences between the treatments (*P<*0.05).

To analyse whether elevated [CO_2_] affects a necrotrophic fungus differently, detached tomato leaves from 5-week-old tomato plants were inoculated with spores of *B. cinerea*. In the comparative assay, elevated [CO_2_]-treated leaves seemed to be much more susceptible to *B. cinerea* than ambient [CO_2_]-treated leaves because a considerably larger increase in the spread of *B. cinerea* lesions was observed at 2 dpi (Fig. 3A). Moreover, a whole-plant inoculation protocol was set up to obtain a more reliable and reproducible system for infection studies than those based on detached leaves (Fig. 3B, C). An analysis of symptom appearance, ΦPSII, and *B. cinerea*-specific actin genes in the whole-plant inoculation experiment again revealed increased infection in elevated [CO_2_]-treated plants.

### Induction of SA- and JA-dependent pathways in different plant–pathogen interactions under elevated [CO_2_]

**Fig. 4.**
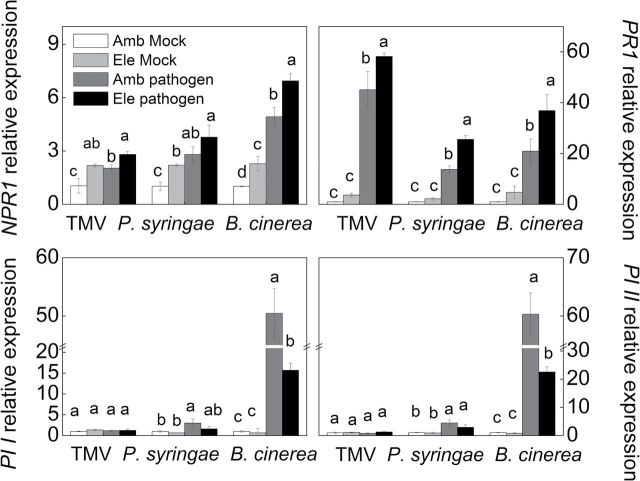
Expression levels of target genes in tomato plants grown under elevated (Ele, 800 μmol mol^–1^) or ambient [CO_2_] (Amb, 380 μmol mol^–1^) with or without inoculation with tobacco mosaic virus, *Pseudomonas syringae*, or *Botrytis cinerea*. Plants with TMV infections were sampled 6 days post-inoculation (dpi), whereas plants with *P. syringae* or *B. cinerea* inoculation were sampled at 2 dpi. The results are expressed as the mean values±SD, *n*=4. Different letters indicate significant differences between the treatments with same pathogen (*P*<0.05).

Whether the tomato plant resistance to different pathogens under elevated [CO_2_] was related to the alterations in the SA/JA defence pathway was then tested. The SA effect occurs mainly through the co-activator NPR1 and pathogenesis-related gene 1 (*PR1*) (Durrant and Dong, 2004; Pieterse and Van Loon, 2004), whereas the JA effect occurs mainly through two JA-dependent genes, *PI I* and *PI II* (AbuQamar *et al.*, 2008). The only *PR1* gene in *Arabidopsis* is a good marker for SA signalling, whereas there are several *PR1* genes in tomato plants; the expression induction of tomato *PR1* gene (accession AK324060.1) used in this study was SA-dependent and JA-independent (Supplementary Fig. S1). The expression levels of these SA- and JA-dependent genes were then detected under different pathogen inoculation and CO_2_ treatment conditions using qRT-PCR (Fig. 4). In mock-inoculated plants, elevated [CO_2_] increased the transcript level of *NPR1* by approximately 117.1% in all three independent experiments with TMV, *P. syringae*, and *B. cinerea*. Similarly, *PR1* expression was also higher in mock-infected elevated [CO_2_]-treated plants; however, no significant quantitative changes were observed. In contrast, the CO_2_ concentration had no effect on *PI I* and *PI II* transcript expression. Pathogen infection significantly increased the SA-dependent gene *NPR1* regardless of the invader type; moreover, pathogen infection induced significantly higher *NPR1* transcripts under elevated [CO_2_] than under ambient [CO_2_]. The *PR1* induction pattern was similar to that of *NPR1*. Conversely, TMV and *P. syringae* infection had little or no effects on JA-dependent *PI I* and *PI II* transcript expression, whereas *B. cinerea* greatly induced *PI I* and *PI II* transcript expression by 50.4- and 60.3-fold under ambient [CO_2_] and by 15.7- and 22.5-fold under elevated [CO_2_], respectively. It should be noted that the increases in *B. cinerea*-induced *PI I* and *PI II* transcript expression were much lower in plants under elevated [CO_2_] than in those under ambient [CO_2_].

**Fig. 5.**
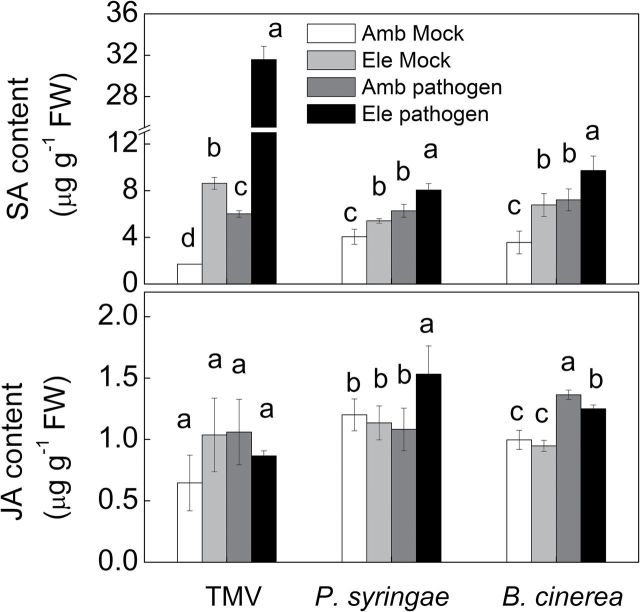
Endogenous phytohormone concentrations in tomato plants grown under elevated (Ele, 800 μmol mol^–1^) or ambient [CO_2_] (Amb, 380 μmol mol^–1^) condition with and without inoculation with tobacco mosaic virus, *Pseudomonas syringae*, or *Botrytis cinerea*. Plants with TMV infections were sampled 6 days post-inoculation (dpi), whereas plants with *P. syringae* or *B. cinerea* inoculation were sampled at 2 dpi. The results are expressed as the mean values±SD, *n*=4. Different letters indicate significant differences between the treatments with same pathogen (*P*<0.05).

The changes of SA and JA accumulation due to different pathogens under elevated [CO_2_] were also determined through HPLC-MS/MS analysis using labelled internal standards (Fig. 5). Elevated [CO_2_] caused a significant increase of SA accumulation in mock plants. TMV, *P. syringae* and *B. cinerea* inoculation raised SA content by 3.5-, 1.5-, and 2.0-fold respectively, under ambient [CO_2_], which were further increased to 18.6-, 2.0-, and 2.7-fold respectively, under elevated [CO_2_]. By contrast, JA content was not significantly affected under elevated [CO_2_] in mock plants. There were also no changes of JA content in TMV or *P. syringae*-inoculated plants under ambient [CO_2_]. Notably, *B. cinerea* inoculation induced JA accumulation by 36.9%, whereas this *B. cinerea*-induced JA content increase was only 25.5% under elevated [CO_2_]. It was unexpected that under elevated CO_2_ condition, *P. syringae* inoculation significantly induced JA content.

### Impairment in SA or JA signalling and biosynthesis affected tomato–pathogen interactions under elevated [CO_2_]

**Fig. 6.**
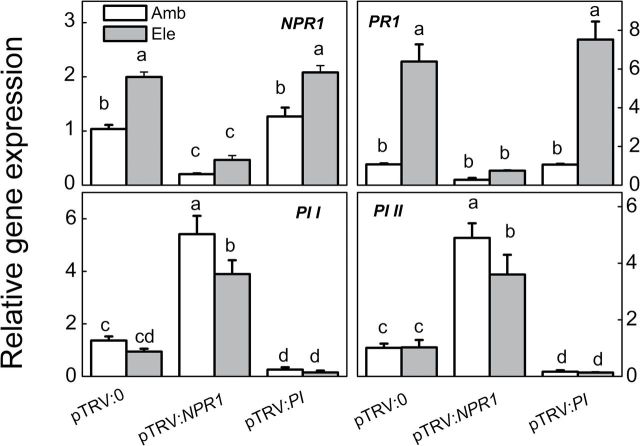
Expression levels of target genes in gene-silenced tomato plants grown under elevated (Ele, 800 μmol mol^–1^) or ambient [CO_2_] (Amb, 380 μmol mol^–1^). Three weeks after seedling inoculation with pTRV:*NPR1*, pTRV:*PI*, or the empty vector pTRV:0, plants were subjected to elevated or ambient [CO_2_] for 5 d, and leaf samples were then collected for gene expression analysis. The results are expressed as the mean values±SD, *n*=4. Different letters indicate significant differences between the treatments (*P*<0.05).

There were significantly different responses of SA- and JA-dependent genes and synthesis to pathogens and CO_2_ conditions. To test whether these responses have a biological effect on plant–pathogen interactions under elevated [CO_2_], VIGS experiments were performed using the TRV vectors. After 3–4 weeks, the *NPR1*, *PR1*, *PI I*, and *PI II* transcript expression levels were analysed in silenced plants (Fig. 6). The transcript expression levels for SA-dependent *NPR1* and *PR1* but not JA-dependent *PI I* and *PI II* were again significantly increased in pTRV:0 plants grown under elevated [CO_2_] compared with those grown under ambient conditions. pTRV:*NPR1*-silenced plants showed significantly lower levels of *NPR1* and *PR1* transcripts in both ambient and elevated [CO_2_] conditions, whereas pTRV:*PI*-silenced plants exhibited significantly reduced *PI I* and *PI II* transcript expression compared with pTRV:0-silenced plants. The experiments were extended to ask whether NPR1 regulates the expression of *PI I* and *PI II* and *vice versa*. It was found that *NPR1* gene silencing resulted in large and significant increases in *PI I* and *PI II* transcript expression under both ambient and elevated [CO_2_] conditions. However, pTRV:*PI*-silenced plants did not exhibit altered levels of *NPR1* and *PR1* transcript expression compared with pTRV:0-silenced plants (Fig. 6).

**Fig. 7.**
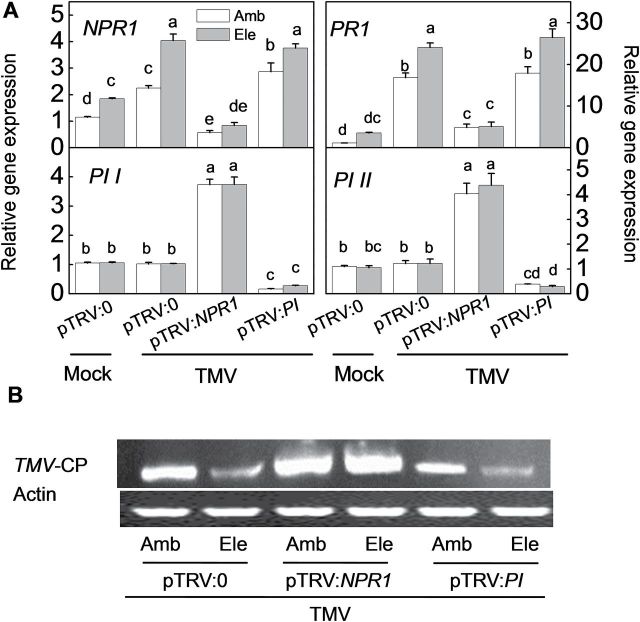
Effects of tobacco mosaic virus infection on gene-silenced tomato plants grown under elevated (Ele, 800 μmol mol^–1^) or ambient [CO_2_] (Amb, 380 μmol mol^–1^). Three weeks after seedling inoculation with pTRV:*NPR1*, pTRV:*PI*, or the empty vector pTRV:0, the plants were subjected to elevated or ambient [CO_2_], with or without TMV inoculation. (A) Expression levels of target genes at 6 days post-inoculation (dpi). The results are expressed as the mean values±SD, *n*=4. Different letters indicate significant differences between the treatments (*P*<0.05). (B) Semi-quantitative analysis of the gene encoding the TMV-coat protein (CP) in young, fully expanded leaves at 9 dpi.

Plants in which genes of interest were silenced were subjected to TMV challenge, and samples were harvested for RNA extraction at 9 dpi. In contrast with mock-inoculated pTRV:0 plants, TMV- and elevated [CO_2_]-induced activation of both *NPR1* and *PR1* was abolished in *NPR1*-silenced plants, whereas these genes were expressed at similar levels in *PI*-silenced plants (Fig. 7A). In contrast, the expression levels of *PI I* and *PI II* did not change in response to TMV or elevated [CO_2_] in pTRV:0 plants, although the expression levels were significantly reduced by silencing *PI*. These transcripts were significantly induced in *NPR1*-silenced plants compared with pTRV:0-silenced plants, suggesting that *NPR1* induction suppresses the expression of the JA-dependent genes *PI I* and *PI II*.

**Fig. 8.**
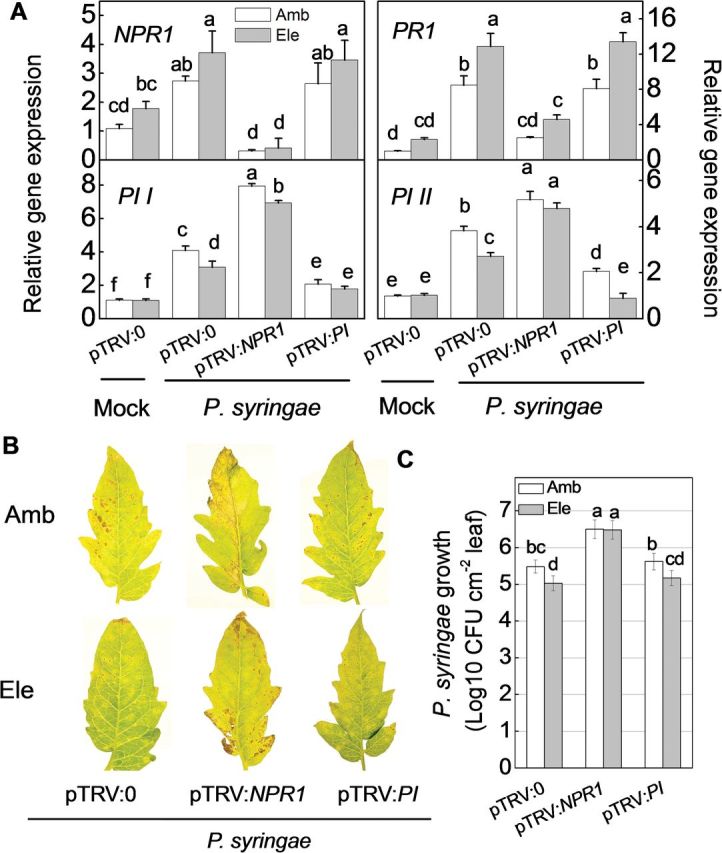
Effects of *Pseudomonas syringae* infection on gene-silenced tomato plants grown under elevated (Ele, 800 µmol mol^–1^) or ambient [CO_2_] (Amb, 380 µmol mol^–1^). Three weeks after seedling inoculation with pTRV:*NPR1*, pTRV:*PI*, or the empty vector pTRV:0, the plants were subjected to elevated or ambient CO_2_ concentrations, with or without *P. syringae* inoculation. (A) Expression levels of target genes at 2 days post-inoculation (dpi). (B) Disease symptoms were photographed at 6 dpi. (C) *In planta* multiplication of *P. syringae* bacterial populations at 4 dpi. The results in A and C are expressed as the mean values±SD, *n*=4. Different letters indicate significant differences between the treatments (*P*<0.05).

**Fig. 9.**
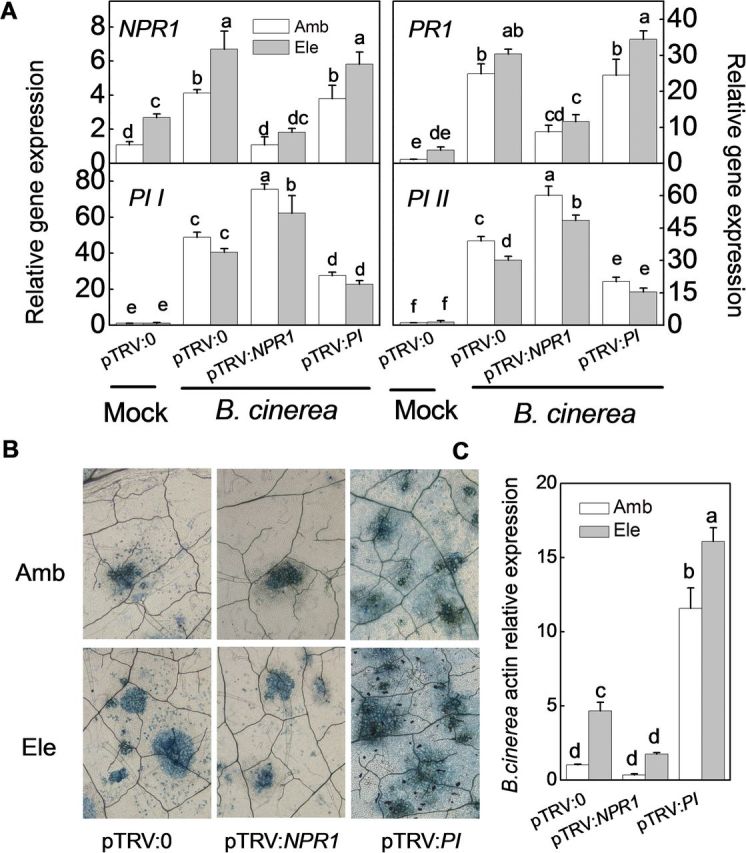
Effects of *Botrytis cinerea* infection on gene-silenced tomato plants grown under elevated (Ele, 800 μmol mol^–1^) or ambient [CO_2_] (Amb, 380 μmol mol^–1^). Three weeks after seedling inoculation with pTRV:*NPR1*, pTRV:*PI*, or the empty vector pTRV:0, the plants were subjected to elevated or ambient [CO_2_], with or without *in planta B. cinerea* spray inoculation. (A) Expression levels of target genes at 2 days post-inoculation (dpi). (B) Trypan blue staining for cell death was performed at 3 dpi. (C) *B. cinerea actin* gene expression at 3 dpi. The results in A and C are expressed as the mean values±SD, *n*=4 in A and *n*=5 in C. Different letters indicate significant differences between the treatments (*P*<0.05).

The changes in SA and JA signalling were further investigated upon challenge with *P. syringae* and *B. cinerea* 2 dpi in response to CO_2_ elevation (Figs 8A and 9A). The transcript abundance differences of SA- and JA- dependent genes were similar between pTRV:0 and gene-silenced plants regardless of the pathogen type. In pTRV:*NPR1*-silenced plants, *NPR1* and *PR1* transcripts were suppressed, whereas *PI I* and *PI II* transcripts were induced compared with those pTRV:0 plants (Figs 8A and 9A). In pTRV:*PI*-silenced plants, the expression levels of *PI I* and *PI II* were significantly reduced, without evident effect on *NPR1* and *PR1* expression (Figs 8A and 9A). Furthermore, regardless of the pathogen invader or the gene silencing constructs, *NPR1* and *PR1* transcript levels were generally higher, whereas *PI I* and *PI II* transcript levels were generally lower, in elevated [CO_2_]-treated plants compared with ambient-treated plants, even though no significant quantitative changes between treatments were observed in some cases (Figs 8A and 9A).

Silencing *NPR1* or *PI* affected tomato–pathogen interactions under elevated [CO_2_] in different ways (Figs 7–9). pTRV:*NPR1*-silenced plants accumulated much more *TMV-CP* RNA than pTRV:0 and pTRV:*PI* plants, regardless of the [CO_2_] conditions. The elevated [CO_2_]-induced TMV resistance was completely abolished in pTRV:*NPR1*-silenced plants (Fig. 7B). These results suggest that elevated [CO_2_]-induced tomato resistance against TMV was associated with SA-dependent NPR1 and PR1. The changes in disease development in response to elevated [CO_2_] and gene silencing were similar between plants inoculated with *P. syringae* and TMV. The symptoms of *P. syringae* infection were much more severe in pTRV:*NPR1*-silenced plants than in pTRV:0 plants, which showed classic symptoms of susceptibility at 6 dpi, such as chlorosis, water-soaked lesions, and necrotic pits (Fig. 8B). The elevated [CO_2_]-induced resistance was also abolished in these plants. In contrast, the pTRV:*PI*-silenced plants did not exhibit altered resistance to *P. syringae* in either ambient or elevated [CO_2_] conditions. Furthermore, bacterial colony numbers were also used to determine the bacterial growth after different treatments, and the *P. syringae* growth results at 4 dpi were in good agreement with the disease symptom (Fig. 8C). Tomato plants were then challenged with *B. cinerea*. As shown in Fig. 9B and C, elevated [CO_2_]-treated plants were more susceptible to *B. cinerea* compared with those grown under ambient conditions. *NPR1* silencing induced resistance to *B. cinerea*, as the cell death and *B. cinerea* actin gene expression were significantly reduced in plants grown under elevated [CO_2_], although these genes did not show significant quantitative differences compared with their expression levels in plants grown under ambient conditions. In contrast, silencing *PI* greatly increased tomato susceptibility to *B. cinerea*, especially under elevated [CO_2_]. To verify the involvement of SA and JA in the variation of disease susceptibility under elevated [CO_2_], tomato genotypes were used that are impaired in their ability to accumulate these hormones, i.e. *NahG* plants defective in SA biosynthesis, *spr2* mutants affected in JA biosynthesis, and their respective wild-type MM and CM. The disease expression of these plants challenged with TMV, *P. syringae*, or *B. cinerea* were monitored, and similar trends were found as the results obtained from VIGS *NPR1* and *PI* plants (Supplementary Fig. S3).

## Discussion

The assessment of plant disease under elevated [CO_2_] conditions is a key step in the development of plant–pathogen management, but experimental research results are often inconsistent (Chakraborty and Datta, 2003; Hoye *et al.*, 2003; McElrone *et al.*, 2005; Lake and Wade, 2009; Pangga *et al.*, 2011; West *et al.*, 2012; Juroszek and von Tiedemann, 2013). Here, reductions in plant disease caused by TMV and *P. syringae* but increases in the incidence and severity of disease caused by *B. cinerea* under elevated [CO_2_] in tomato plants were documented. Endogenous hormone biosynthesis, transcripts of genes involved signalling, and gene silencing/mutant experiments provided evidence that the variation in disease susceptibility is potentially related to flexibility in leaf chemistry and cross talk between the SA and JA signalling pathways. Therefore, these results are critical for understanding the effects of elevated [CO_2_] on plant–pathogen microbe interactions and will help to ameliorate the negative effects and to use the benefits of elevated [CO_2_] in managed agricultural and natural ecosystems.

The effects of elevated [CO_2_] on plant–pathogen interactions are expected to occur both directly through plant physiological responses and indirectly through effects on microbes that associate with plants (Malmstrom and Field, 1997; Rua *et al.*, 2013). The *in vitro* pathogen microbe growth studies clearly showed that the bacteria *P. syringae* and the necrotrophic fungus *B. cinerea* were not affected by elevated [CO_2_] (Supplementary Fig. S2). Similarly, based on the studies of plant pathogens *Erwinia* spp. and *Pseudomonas fluorescens*, Wells (1974) observed no inhibitory effects on cell growth in liquid culture from 0.03–3% CO_2_. Thus, the most pronounced effects of elevated [CO_2_] are on host physiology. We thus speculated that changes in plant physiology, i.e. biochemical profiles of pathogen-infected plants under elevated [CO_2_], may result in increased resistance or susceptibility to specific pathogens (Matros *et al.*, 2006). In the current study, the involvement of the phytohormones SA and JA was examined under elevated [CO_2_] with and without pathogen inoculation. Regardless of the pathogen type, elevated [CO_2_] generally increased constitutive levels of SA and SA-related transcripts in both uninfected and infected plants, especially in the additive treatments of elevated [CO_2_] and pathogen infection (Figs 4 and 5). In contrast to the universal increase in SA, JA concentrations and the transcripts of genes involved in JA signalling were not increased by elevated [CO_2_] in uninfected plants. TMV and *P. syringae* infection had little or no effects on the JA contents or transcripts of genes involved in JA signalling, although these genes were induced by *B. cinerea*. Furthermore, the *B. cinerea*-induced increases in JA contents as well as *PI I* and *PI II* transcript levels were much lower in plants grown under elevated [CO_2_] compared with those grown under ambient [CO_2_] (Figs 4 and 5). These results suggest that elevated [CO_2_] favours the SA pathway but represses the JA pathway in plants. Similar results have been observed in other studies using tomato and soybean plants (Zavala *et al.*, 2008; Sun *et al.*, 2011; Huang *et al.*, 2012; Zavala *et al.*, 2013).

Thus, whether the elevated [CO_2_]-induced variation in hormonal signalling was associated with the observed variation in the tomato defence against different pathogens under elevated [CO_2_] was investigated. Specifically, in this study, under elevated [CO_2_], disease caused by TMV and hemibiotrophic *P. syringae* decreased, whereas plant disease incidence and severity caused by necrotrophic *B. cinerea* significantly increased compared with ambient [CO_2_] (Figs 1–3). It has been accepted that SA signalling is generally important for defence against biotrophs or hemibiotrophs such as *P. syringae*, whereas JA signalling generally is relevant for defence responses directed against necrotrophs, although there are exceptions (Tsuda and Katagiri, 2010; Kliebenstein, 2014). In this study, the basal defence against TMV and the bacterial pathogen *P. syringae* was reduced in *NPR1*-silenced and *NahG* plants, which have altered SA signalling and biosynthesis, but not in *PI*-silenced or *spr2* plants. The converse trend was observed in plants treated with the fungal necrotrophic pathogen *B. cinerea* (Figs 7–9; Supplementary Fig. S3). These results verify the prediction made by previous studies that the basal defence against TMV and *P. syringae* is SA-dependent whereas the defence against *B. cinerea* is controlled by JA signalling (Thomma *et al.*, 1998; Kunkel and Brooks, 2002; Ton *et al.*, 2002). Furthermore, silencing *NPR1* not only abolished elevated [CO_2_]-induced TMV and *P. syringae* resistance but also alleviated elevated [CO_2_]-induced *B. cinerea* susceptibility. In contrast, silencing *PI* further enhanced *B. cinerea* susceptibility under both ambient or elevated [CO_2_], but it had no evident effects on resistance to TMV or *P. syringae* (Figs 7–9). These observations were in accordance with the experiment using *NahG* and *spr2* plants (Supplementary Fig. S3). These results suggest that elevated [CO_2_]-induced tomato defence against TMV, *P. syringae*, and *B. cinerea* was associated with SA/JA signalling cross talk. Elevated [CO_2_] favours SA signalling, leading to resistance to TMV and *P. syringae*, while dampening the JA-related defence against *B. cinerea*. Accordingly, previous studies indicated that the accumulation of SA is often negatively correlated with the accumulation of JA and JA pathway-specific defences (Stout *et al.*, 2006). It has been suggested that the induction of the SA signalling pathway suppresses JA-responsive gene expression downstream or upstream of jasmonate biosynthesis (Doares *et al.*, 1995; Leon-Reyes *et al.*, 2010). In *Arabidopsis*, SA-mediated suppression of JA accumulation and JA-induced defence gene expression is blocked in *npr1* mutants, demonstrating a crucial role for NPR1 in the cross talk between SA and JA signalling (Spoel *et al.*, 2007). In this study, the increased *NPR1* transcripts under elevated [CO_2_] may be responsible for down-regulating JA-related defences and increasing susceptibility to *B. cinerea* in plants grown under CO_2_ enrichment (Figs 4, 6 and 9). Alternatively, NPR1 might also be post-transcriptionally regulated by elevated [CO_2_], which could then inhibit JA-induced defence gene expression (Casteel *et al.*, 2012). At this time, the mechanism by which elevated [CO_2_] alters hormonal responses is unclear. Along with the direct effect of elevated [CO_2_] on plant physiology and growth, elevated [CO_2_] may cause plants to re-allocate resources to synthesize secondary metabolites, which might contribute to SA synthesis and SA/JA cross talk (Matros *et al.*, 2006; Runion *et al.*, 2010). Previous studies have also suggested that elevated [CO_2_] induces SA accumulation and that NPR1 may be activated by the altered redox status in the cytosol through increased thioredoxin and glutathione-*S*-transferase production (Casteel *et al.*, 2012; Zavala *et al.*, 2013). In view of the potential for cross talk between the SA and JA signalling pathways, it might be expected that the elevated [CO_2_]-induced accumulation of SA is related to the suppression of JA signalling, which underlies the variation in plant defences against different pathogen types under elevated [CO_2_]. It should be noted that the cross talk between the JA and SA signalling pathways might also be modified by the pathogens infection to some extent, as expression induction of *PI I* and *PI II* by *NPR1* silencing were generally lower in elevated [CO_2_] compared with ambient [CO_2_], in normal control, *P. syringae-*, or *B. cinerea*-inoculated plants (Figs 6, 8, 9). However, no such difference was observed in the different [CO_2_] when plants are TMV infected.

Few previous studies have attributed SA/JA cross talk to elevated [CO_2_]-induced plant pathogen defences by investigating interactions between pathogens with different infection strategies in the same system. Whether the impact of elevated [CO_2_] on SA/JA cross talk and the associated pathogen defences is a general response is an open question. Many previous studies have reported that under elevated [CO_2_] conditions, lower levels of disease are caused by biotrophic pathogens such as downy mildew caused by *Peronospora manshurica* on soybean (Eastburn *et al.*, 2010) and virus disease caused by potato virus Y on tobacco (Matros *et al.*, 2006). Conversely, some studies have reported that higher levels of disease under elevated [CO_2_] conditions are caused by necrotrophic pathogens such as brown spot caused by *Septoria glycines* (Eastburn *et al.*, 2010) and powdery mildew caused by *Podosphaera xanthii* on zucchini (Pugliese *et al.*, 2011). However, there are also examples of necrotrophic, biotrophic, and hemibiotrophic pathogens having reduced, increased, or no effects on disease upon increased [CO_2_] (Lake and Wade, 2009; Eastburn *et al.*, 2011; Oehme *et al.*, 2013). It should be noted that *B. cinerea* disease was still higher in pTRV:*NPR1* and pTRV:*PI*-silenced plants under elevated [CO_2_] than under ambient conditions (Fig. 9); *NahG* and *spr2* plants also showed similar trend (Supplementary Fig. S3). These might be explained by the other hormone player(s). ET has been shown to act synergistically with JA in the response to *B. cinerea* in *Arabidopsis* (Thomma *et al.*, 1999). In tomato plants, JA-mediated responses seem to act independently from ethylene-induced resistance against *B. cinerea*, and plants pre-treated with ethylene showed a decreased susceptibility toward *B. cinerea*, whereas pre-treatment with 1-methylcyclopropene (an inhibitor of ethylene perception), resulted in increased susceptibility (Díaz *et al.*, 2002). Furthermore, previous studies also indicated that elevated CO_2_ suppresses the ethylene signalling pathway in soybean and *Medicago truncatula* (Zavala *et al.*, 2009; Guo *et al.*, 2014). Thus, ET might be involved in the susceptibility to *B. cinerea* under elevated [CO_2_]. Additionally, in a previous study with *Arabidopsis*, elevated [CO_2_] attenuated the SA-dependent runaway cell death in *lesion simulating disease* 1 (*lsd1*) mutant, which has been implicated in defence following avirulent or virulent pathogen challenge (Mateo *et al.*, 2004). Given the complexity of the interactions between plants, plant pathogens, and the environment, it is not surprising that the understanding of how elevated [CO_2_] influences plant pests and disease agents is still incomplete and requires further study.

**Fig. 10.**
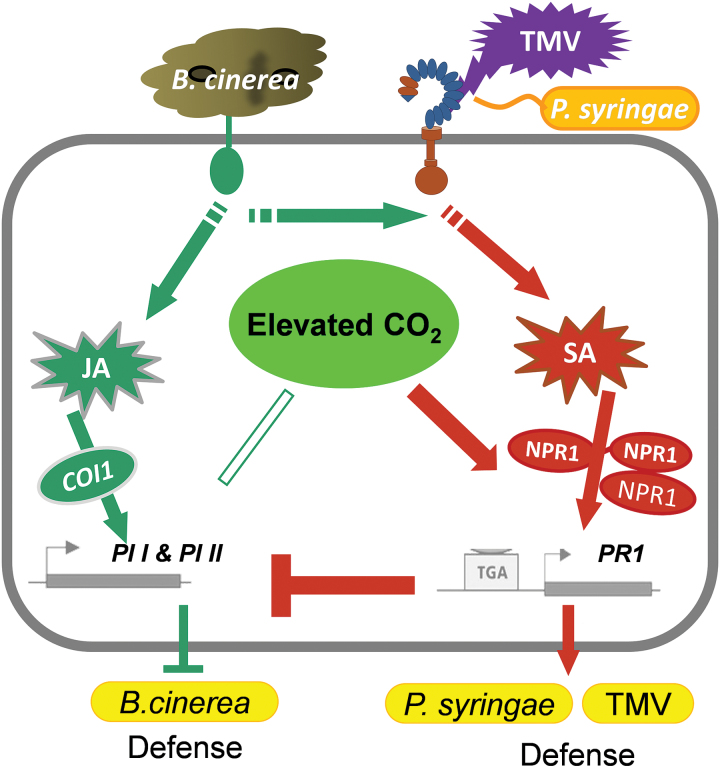
A hypothetical model for the role of phytohormone cross talk in the variation of the defences to plant pathogens under elevated CO_2_ concentrations. Elevated [CO_2_] up-regulated SA synthesis and signalling, including the *PR1* and *NPR1* genes, but it did not up-regulate components of the JA pathway. Thus, the altered SA/JA cross talk favoured the SA pathway-dependent defence but repressed the JA pathway-dependent defence, leading to reductions in plant disease caused by tobacco mosaic virus and *Pseudomonas syringae*, but increases in *Botrytis cinerea* incidence and severity under elevated [CO_2_] in tomato plants.

In conclusion, these results support the hypothesis that the variation in plant disease susceptibility under elevated [CO_2_] is related to cross talk between the SA and JA signalling pathways (Fig. 10). Elevated [CO_2_] up-regulated SA synthesis and signalling, and increased *PR1* and *NPR1* expression, but it did not up-regulate components of the JA pathway. Thus, the altered SA/JA cross talk favours SA pathway-dependent defence but represses JA pathway-dependent defence, leading to a reduction in plant disease susceptibility to TMV and *P. syringae* but an increase in *B. cinerea* incidence and severity under elevated [CO_2_] in tomato plants. This work highlight the modulated antagonistic relationship between SA and JA that contributes to the variation in disease susceptibility under elevated [CO_2_]. These findings lead to the prediction that plants will experience increased resistance to some pathogens and increased susceptibility to others in the future when CO_2_ concentrations increase. Furthermore, the variation in the response to elevated [CO_2_] in plants suggests the potential for phytohormone signalling and defences to serve as targets for breeding efforts and disease management strategies upon climate change in elevated-[CO_2_] agronomic ecosystems.

## Supplementary data

Supplementary data are available at *JXB* online

Figure S1. Effects of exogenous salicylic acid (SA) and methyl jasmonate (MeJA) application on *PR1* gene expression.

Figure S2. *In vitro* pathogen growth in elevated or ambient [CO_2_] concentrations.

Figure S3. Effects of pathogens inoculation on disease expression of wild-type, SA-, and JA-deficient tomato plants under elevated or ambient [CO_2_].

Table S1. Primers used in this study.

Supplementary Data

## Abbreviations:

B. cinerea: *Botrytis cinerea*
dpi: days post-inoculation
FACE: free-air CO_2_ enrichment
JA: jasmonic acid
NPR1: nonexpressor of pathogenesis-related genes 1
PI I and PI II: proteinase inhibitors I and II
PR1: pathogenesis-related protein 1
P. syringae: *Pseudomonas syringae*
PSII: photosystem II
SA: salicylic acid
TMV: tobacco mosaic virus
ΦPSII: photochemical quantum yield at photosystem II.
