# Supplementary material for: Antagonism between phytohormone signalling underlies the variation in disease susceptibility of tomato plants under elevated CO2
Source: J Exp Bot. 2015 Feb 5;66(7):1951–63. doi: 10.1093/jxb/eru538 (PMC4378629; doi:10.1093/jxb/eru538)
Supplement: Supplementary Data [file supp_66_7_1951__index.html]

Antagonism between phytohormone signalling underlies the variation in disease susceptibility of tomato plants under elevated CO2 — Antagonism between phytohormone signalling underlies the variation in disease susceptibility of tomato plants under elevated CO2 — Supplementary Data 

# Antagonism between phytohormone signalling underlies the variation in disease susceptibility of tomato plants under elevated CO2

## Supplementary Data

Data files

**Files in this Data Supplement:**

- Supplementary Data - Supplementary Data
