# Supplementary material for: Antagonism between phytohormone signalling underlies the variation in disease susceptibility of tomato plants under elevated CO2
Source: J Exp Bot. 2015 Feb 5;66(7):1951–63. doi: 10.1093/jxb/eru538 (PMC4378629; doi:10.1093/jxb/eru538)
Supplement: Supplementary Data [file supp_eru538_jexbot139469_file001.pdf]

Journal of Experimental Botany

Antagonism between phytohormone signaling underlies the variation in disease susceptibility of tomato plants under elevated CO_2

Shuai Zhang, Xin Li, Zenghui Sun, Shujun Shao, Lingfei Hu, Meng Ye, Yanhong Zhou, Xiaojian Xia, Jingquan Yu and Kai Shi

SUPPLEMENTARY DATA

Supplementary Figure S1

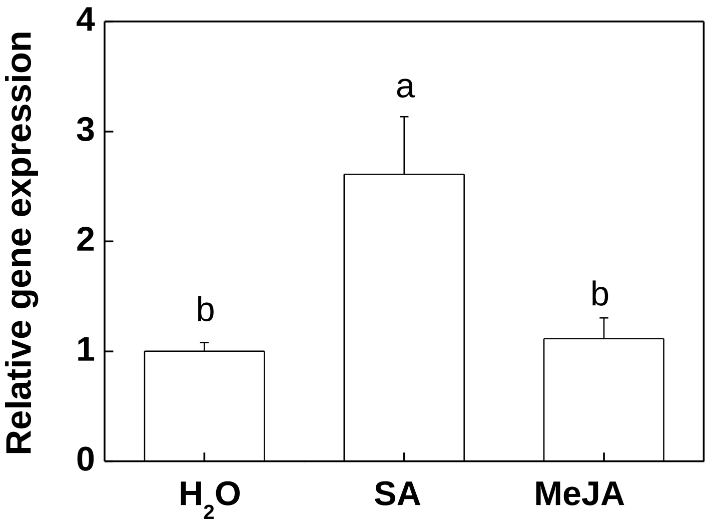

Figure S1 Effects of exogenous salicylic acid (SA) and methyl jasmonate (MeJA) application on *PR1* gene expression. Tomato leaves were collected at 8 h after SA (1 mM) or MeJA (100 µM) spray treatment. The results are presented as mean values ± standard deviation, n = 4. Different letters indicate significant differences between treatments ($P < 0.05$).

Supplementary Figure S2

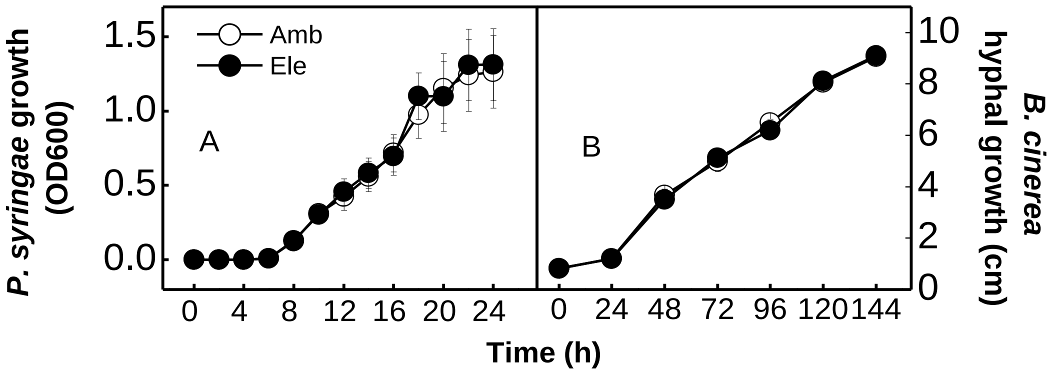

Figure S2. *In vitro* pathogen growth in elevated (800 μmol mol^{-1}) or ambient [CO_2] (380 μmol mol^{-1}). (A) *Pseudomonas syringae* growth rate in King's B liquid medium. (B) *Botrytis cinerea* hyphal growth rate in V8 agar solid media. The results are expressed as the mean values ± SD, *n* = 10.

Supplementary Figure S3

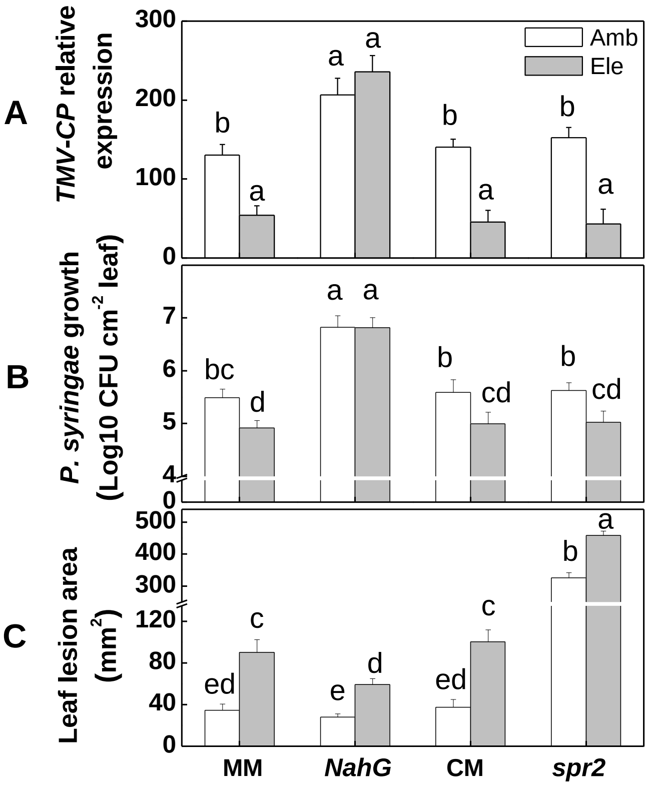

Figure S3. Effects of pathogens inoculation on disease expression of wild-type, SA-, and JA-deficient tomato plants under elevated (Ele, 800 μmol mol^{-1}) or ambient [CO_2] (Amb, 380 μmol mol^{-1}). (A) Transcription of the gene encoding the *Tobacco mosaic virus*-coat protein (TMV-CP) in young, fully expanded leaves at 9 days post TMV inoculation on the lower leaves. (B) *In planta* multiplication of *Pseudomonas syringae* bacterial populations at 4 days post *P. syringae* inoculation. (C) Leaf lesion area at 4 days post *in vitro* inoculation with *Botrytis cinerea* on the detached leaves. For tomato genotypes, wild-type Moneymaker (MM) and its SA-deficient *NahG* transgenic plants, wild-type Castlemart (CM) and its JA-deficient *spr2* mutant plants were used. The results are expressed as the mean values ± SD, n = 4. Different letters indicate significant differences between the treatments ($P < 0.05$).

Supplementary Table S1. Primers used in this study.

Gene	Encoded protein	Accession No	Primer pair	Assay
SlActin	*Solanum lycopersicum* Actin	U60481.1	F: 5’-TGGTCGGAATGGGACAGAAG-3’<br>R: 5’-CTCAGTCAGGAGAACAGGGT-3’	qPCR
TMV-CP	*Tobacco mosaic virus* (U1) coat protein	V01408.1	F: 5’-TTCTTGTCATCAGCGTGGGC-3’<br>R: 5’-TTCGGCAGTCGTGGGGTTC-3’	qPCR & Semi-qPCR
B. cinerea Actin	*Botrytis cinerea* Actin	AY647218	F: 5’-GGTAACATTGTTATGTCTGG-3’<br>R: 5’-CTTGACCTTCATCGACG-3’	qPCR
NPR1	*Nonexpressed Pathogen Related 1*	AY640378	F: 5’-GGGAAAGATAGCAGCACG-3’<br>R: 5’-GTCCACACAAACACACACATC-3’	qPCR
			F: 5’- CGgaattcACTTCTTCGCTGATGCTAAGC-3’<br>R: 5’- CGggatccGACCACGGCATCAAAACTCACC-3’	VIGS
			F: 5’-GGGAAAGATAGCAGCACG-3’<br>R: 5’-GTCCACACAAACACACACATC-3’	VIGS tests
PR1	*Pathogenesis-Related protein 1*	AK324060.1	F: 5’-ATCTCATTGTTACTCACTTGTC-3’<br>R: 5’-AACGAGCCCGACCA-3’	qPCR
PI I	*Proteinase Inhibitors I*	K03290	F: 5’-GAAGTAATTAAGCAGCCACAATATG-3’<br>R: 5’-GCCCCCCTTATTTTTTCC-3’	qPCR
			F: 5’- CGgaattcGACTTCTTCGCTGATGCTAAGC-3’<br>R: 5’- CGggatccGACCACGGCATCAAAACTCACC-3’	VIGS
			F: 5’-GAAGTAATTAAGCAGCCACAATATG-3’	VIGS tests

			R: 5’-GCCCCCCTTATTTTTTCC-3’	
PI II	Proteinase Inhibitors II	K03291.1	F:5’-CCTATTCAAGATGTCCCCGTTC-3’<br>R:5’-GGGCAATCCAGAAGATGG-3’	qPCR
			F: 5’-CGgaattcATGGCTGTTCACAAGGAAGTTAATTTTGTC-3’<br>R: 5’-CGggatccTCACATTACAGGGTACATATTTGCCTTGGG-3’	VIGS test
			F: 5’-CCTATTCAAGATGTCCCCGTTC-3’<br>R: 5’-GGGCAATCCAGAAGATGG-3’	VIGS tests
